# Microbiota of Chicken Breast and Thigh Fillets Stored under Different Refrigeration Temperatures Assessed by Next-Generation Sequencing

**DOI:** 10.3390/foods10040765

**Published:** 2021-04-03

**Authors:** Dimitra Dourou, Evgenia D. Spyrelli, Agapi I. Doulgeraki, Anthoula A. Argyri, Athena Grounta, George-John E. Nychas, Nikos G. Chorianopoulos, Chrysoula C. Tassou

**Affiliations:** 1 Institute of Technology of Agricultural Products, Hellenic Agricultural Organization DIMITRA, Sofokli Venizelou 1, Lycovrissi, 14123 Athens, Greece; ddourou@hotmail.com (D.D.); adoulgeraki@aua.gr (A.I.D.); anthi.argyri@gmail.com (A.A.A.); athenagrounta@gmail.com (A.G.); nchorian@nagref.gr (N.G.C.); 2 Laboratory of Food Microbiology and Biotechnology, Department of Food Science and Human Nutrition, School of Food and Nutritional Sciences, Agricultural University of Athens, Iera Odos 75, 11855 Athens, Greece; eugeniespcheng@gmail.com (E.D.S.); gjn@aua.gr (G.-J.E.N.)

**Keywords:** chicken meat, refrigerated storage, next-generation sequencing (NGS), bacterial communities, microbiological analysis, metagenetic analysis

## Abstract

Chicken is one of the most widely consumed meats worldwide. The exploration of the bacterial diversity of chicken meat may provide new insights into the chicken-associated microbiome that will lead to moderation of food spoilage or safety. This study was undertaken to explore the bacterial communities of chicken breast and thigh fillets stored at refrigeration (0 °C and 5 °C) and slightly abuse (10 °C) temperatures for 5 days through conventional cultural methods along with next-generation sequencing (NGS) analysis. Total viable counts (TVC), *Brochothrix thermosphacta*, *Pseudomonas* spp., and lactic acid bacteria (LAB) were enumerated, while the bacterial communities were mapped through 16S rRNA gene amplicon sequencing. Chicken breast and thigh fillets possessed a complex bacterial structure that incorporated a total of >200 Operational Taxonomic Units (OTUs) at the genus level. The core microbiota of fresh samples consisted of *Acinetobacter*, *Brochothrix*, *Flavobacterium*, *Pseudomonas*, *Psychrobacter*, and *Vibrionaceae* (family). These genera persisted until the end of storage in >80% of samples, except *Psychrobacter* and *Flavobacterium*, while *Photobacterium* was also identified. Hierarchical clustering showed a distinction of samples based on storage time and chicken part. Conventional plate counting with growth media commonly used in spoilage studies did not always correspond to the microbial community profiles derived from NGS analysis, especially in *Pseudomonas*, *Acinetobacter*, *Photobacterium*, and *Vibrionaceae*. Results of the present study highlight *Photobacterium* and *Vibrionaceae*, in general, as potent chicken meat spoilers and suggest the necessity to combine classical microbiological methods along with NGS technologies to characterize chicken meat spoilage microbiota.

## 1. Introduction

Global consumption of poultry meat has rapidly increased over the last few decades and is expected to rise further in the coming decade [1]. The reasons for this remarkable increase include the growing health awareness (i.e., consumer demand for low-fat, high-quality protein meats) and sustainability issues, such as environmental (i.e., reduction of meat industry’s environmental footprint), economic (i.e., affordability, low investment and production cost, short rearing cycle), and social (i.e., absence of cultural or religious constraints limiting consumption) [1,2,3,4]. 

Fresh chicken meat is highly perishable, owing to its physical and chemical properties. Attributes such as high water holding capacity and abundance of nitrogenous compounds, lipids, carbohydrates, and vitamins make chicken meat a favorable environment for microbial growth [5,6]. Whereas the muscles of healthy living birds are sterile, microbial contamination deriving from animal microbiota, processing environment, and human personnel is unavoidably introduced to the carcass and chicken cuts [7,8,9]. The presence and proliferation of microorganisms on meat results in sensorial modifications [10,11] that render chicken products unacceptable for human consumption, usually within a week post-slaughter [7,12,13]. Bacterial communities commonly found on fresh meat are *Acinetobacter*, *Pseudomonas*, *Brochothrix*, *Flavobacterium*, *Psychrobacter*, *Moraxella*, *Staphylococcus*, *Micrococcus*, lactic acid bacteria (LAB), and *Enterobacteriaceae* [5,7,14,15]. However, a fraction of the initial microbiota, the so-called specific spoilage organisms (SSOs), is dominant in food spoilage [10,13]. Initial microbial types and loads on meat muscle, the storage conditions (mainly temperature and availability of oxygen), and the cold chain shape the SSOs selection on raw chicken meat [14]. Despite the tremendous technological advances in food preservation over the years, food spoilage remains a long-lasting global threat that leads to economic losses for the poultry industry and, most importantly, to food loss and waste [11,16]. Therefore, a deep analysis of bacterial communities and structures on chicken during cold storage and the identification of predominant spoilage bacteria is required to minimize occurrences of contamination and spoilage. 

Traditionally, the characterization of microbiota on foods matrices was performed by standard cultivation and phenotyping methods [17]. However, the “gold standard” plate count method underestimates the diversity of complex bacterial communities, since less than 1% of environmental bacteria are practically culturable [18,19,20,21]. The introduction of culture-independent approaches has delivered substantial insights into microbial community profiling in support of traditional techniques. The advances of sequencing technologies in recent years, along with new bioinformatics tools, have evolved DNA sequencing technologies from first (low-throughput) to next (high-throughput) generation [22,23,24]. Next-generation sequencing (NGS) technology allows for exploring bacterial taxa (including nonculturable or in low number bacteria) and their evolution on the chicken meat ecosystem. 

To date, various studies have employed NGS to investigate the chicken microbiome. However, these studies have mainly focused on the chicken gastrointestinal tract [25,26,27,28,29], chicken carcasses at various stages throughout processing line [30,31,32,33,34], and fresh chicken cuts [35,36,37,38]. To our knowledge, limited studies have investigated the overall microbial profile on chicken broiler meat or cuts stored under chilled conditions by amplicon sequencing [34,39,40,41,42]. Characterizing the microbiota of chicken meat during cold storage can expand our understanding of poultry spoilage in a multifaceted and integrative manner.

In this work, the microbiota of chicken breast and thigh fillets stored at refrigerated (0 °C and 5 °C) and slightly abuse (10 °C) temperatures was explored with NGS 16S rRNA gene along with the culture-dependent conventional plating technique. Additionally, an effort was made to characterize the core microbiota encompassing putative spoilers from refrigerated chicken meat and to explore the potential association between certain taxa, chicken part, and/or storage temperature. 

## 2. Materials and Methods

### 2.1. Chicken Cuts Provision and Storage

Chicken breast and thigh fillets were obtained from a local manufacturer in Greece (KOTINO S.A., Nea Artaki, Greece) and transported (within 30 min) to the laboratory under refrigeration. Chicken breast fillets were supplied in plastic packages (width: 25 cm; thickness: 90 μm; permeability of ca. 25 cm³, 90 cm³, and 6 cm³ m^−2^day^−1^bar^−1^ at 20 °C and 50% RH for CO_2_, O_2_, and N_2_, respectively), while thigh fillets were separately placed in Styrofoam trays and manually wrapped with air-permeable polyethylene cling film. This procedure was followed to simulate the packaging used by the industry to distribute these products. Samples were then stored under well-controlled isothermal refrigerated (i.e., 0 °C and 5 °C) and slightly abuse (i.e., 10 °C) temperatures in high-precision (±0.5 °C) programmable incubators (MIR-153, Sanyo Electric Co., Osaka, Japan) to simulate the range of temperatures that could be encountered in the food chain [43]. For each sampling point, 1 sample per each independent batch (2 batches/lot) and from each chicken part (breast and thigh fillets) and storage temperature (0 °C, 5 °C, and 10 °C) was collected. More specifically, 4 samples (2 batches × 2 chicken parts) from day 0 (i.e., fresh samples) and 12 samples (2 batches × 2 chicken parts × 3 storage temperatures) after 5 days of storage (i.e., close to shelf life and simulating consumer practice) were analyzed. 

### 2.2. Microbiological Analyses and pH Measurement

For the enumeration of the main bacterial groups responsible for chicken breast and thigh fillets spoilage, an excision sampling method was undertaken [44,45]. Excision involved removing a 5 cm^2^ sliver of chicken tissue (maximum thickness of 2 mm) from 4 different sites (20 cm^2^ total area) of each breast and thigh fillet with a sterile stainless-steel cork borer. Excised samples were individually diluted with 100 mL of ¼ strength Ringer’s solution (LAB M Limited, Lancashire, United Kingdom) and homogenized for 2 min at room temperature (Lab Blender 400, Seward Limited, London, United Kingdom). Appropriate decimal dilutions of the resulting homogenate were spread- (0.1 mL) or pour- (1 mL) plated on different nonselective and selective media for the enumeration of the following bacterial groups (a) Total viable counts (TVC) on plate count agar (Tryptic Glucose Yeast Agar PCA, Ref. 4021452, Biolife, Italiana S.r.l, Milano, Italy) and incubated at 25 °C for 72 h, (b) *Pseudomonas* spp. on Pseudomonas agar base (LAB108 supplemented with selective supplement Cetrimide Fucidin Cephaloridine, Modified C.F.C X108, LABM) and incubated at 25 °C for 48 h, (c) *Brochothrix* (*B.*) *thermosphacta* on Streptomycin Thallous Acetate Actidione Agar (Ref. 4020792 with the addition of antibiotic REF 4240052, Biolife, Italiana S.r.l, Milano, Italy) incubated at 25 °C for 48 h, and (d) LAB on de Man, Rogosa and Sharpe (MRS) medium (Ref.401728S2, Biolife, Italiana S.r.l, Milano, Italy) overlaid with the same medium and incubated at 30 °C for 72 h. Prior to enumeration, all plates were examined visually for typical colony types and morphological characteristics associated with each growth medium. Additionally, the selectivity of each growth medium was checked by microscopic examination of smears prepared from randomly selected colonies and Gram staining.

The pH value of chicken parts was evaluated with a digital pH meter (RL150, Russell pH, Cork, Ireland) upon completion with the microbiological analyses, by inserting a pH glass electrode (Metrohm AG, Herisau, Switzerland) into the homogenate at room temperature. 

### 2.3. Total Bacterial DNA Extraction 

Chicken breast and thigh fillet homogenates (100 mL) were aseptically transferred to sterile centrifuge tubes and concentrated to ca. 1 mL by centrifugation (10,000× *g*, 4 °C, 10 min, in duplicate) to collect bacterial cells. Cell suspensions from 2 samples (1 sample per batch/lot) per chicken part at the beginning (i.e., fresh samples, day 0) and after 5 days of storage (i.e., close to shelf life and simulating consumer practice) were then pooled into a new sterile 2 mL tube for further analysis. According to the manufacturer’s instructions, isolation of bacterial genomic DNA was performed with the NucleoSpin Food kit (Macherey-Nagel GmbH & Co KG, Düren, Germany) using 200 μg of the cell suspension. DNA quantification and purity testing were performed with the NanoDrop 2000 spectrophotometer (Thermo Fisher Scientific, Walthman, MA, USA).

### 2.4. Microbiome Analysis through Next Generation Sequencing (NGS)

The Ion 16S Metagenomics kit (Thermo Fisher Scientific, A26216) was used to amplify the V2-4-8 and V3-7-9 hypervariable regions of 16S rRNA of genomic DNA isolated from chicken breast and thigh fillets. The resulting amplicons (up to 400 bp) were sequenced using the Ion Torrent PGM by CeMIA SA (https://cemia.eu/, accessed on 15 December 2020) (Larissa, Greece) in order to evaluate the microbial communities. The sequencing results were analyzed with Ion Reporter^TM^ (Thermo Fisher Scientific, Waltman, MA, USA) software, which provides a fast and semiquantitative evaluation of complex microbial samples. Chimeras and noise were removed from the sequences. Operational taxonomic units (OTUs) were taxonomically classified (at >97% similarity) according to Spyrelli et al. [46] using the Nucleotide Basic Local Alignment Search Tool (BLASTn) against the National Centre for CBI database (www.ncbi.nlm.nih.gov, accessed on 15 December 2020) (Bethesda, MD, USA).

### 2.5. Statistics and Multivariate Data Analysis 

The significance of differences in microbial populations and pH values between chicken breast and thigh fillets samples (*n* = 2) stored at different isothermal temperatures were tested with 1-way analysis of variance (ANOVA) followed by Tukey’s multiple range tests at *p* < 0.05. Statistical analysis was performed with SPSS (IBM SPSS Statistics for Windows, Version 26.0. Armonk, NY, USA: IBM 283 Corp.). 

To determine the relationship between chicken parts and given microbiota at the OTU genus level, multivariate statistics using hierarchical cluster analysis and partial least squares-discriminant analysis (PLS-DA) were performed. Validation of the PLS-DA model was performed using 100 permutations [47]. Hierarchical cluster analysis, with Pearson’s correlation coefficient as the similarity measure and Ward’s linkage as the clustering algorithm [48], was additionally performed to visualize relationships among chicken parts and the characterized microbiota. Results were visualized in the form of a dendrogram. The raw data (genus-level OTUs) used were transformed by Pareto scaling before statistical analysis. PLS-DA analysis and hierarchical clustering were performed using MetaboAnalyst 4.0 [49]. Venn diagrams were generated to illustrate unique and shared genera using Venny 2.1.0 [50]. 

## 3. Results 

### 3.1. Microbial Evolution and pH Values 

**Figure 1:**
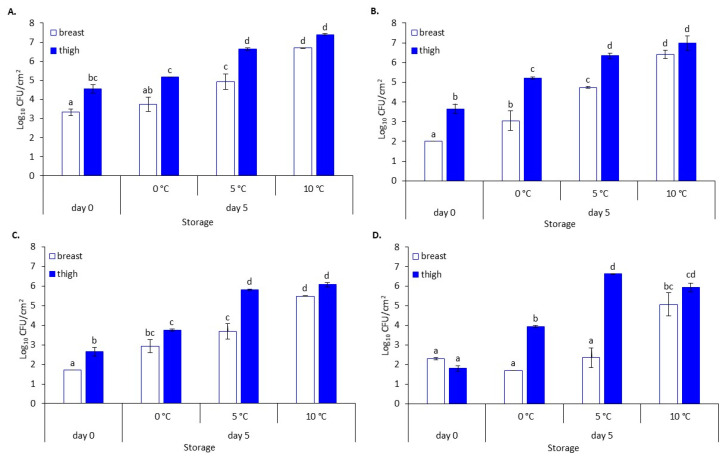
Microbial population (mean ± standard deviation, *n* = 2) of (**A**) total viable counts (TVC), (**B**) *Pseudomonas* spp., (**C**) *B. thermosphacta***,** and (**D**) lactic acid bacteria (LAB) on chicken breast and thigh fillets at the beginning (day 0) and end (day 5) of storage at 0 °C, 5 °C, and 10 °C. Different letters (a–d) indicate statistically (*p* < 0.05) significant differences.

The microbial populations of chicken breast and thigh fillets stored at different refrigerated (0 °C and 5 °C) and slightly abuse (10 °C) temperatures are illustrated in Figure 1. At the beginning of storage, TVC on breast and thigh fillets varied (*p* < 0.05) and were 3.3 ± 0.2 log CFU/cm^2^ and 4.6 ± 0.2 log CFU/cm^2^, respectively. The main bacterial spoilage bacteria consisted mainly of *Pseudomonas* spp., *B. thermosphacta* and LAB. The changes in the aforementioned bacterial groups and their contribution to the final microbiota were influenced by storage time and temperature. At the end of the storage (day 5), TVC ranged from 3.7 ± 0.4 log CFU/cm^2^ to 6.7 ± 0.0 log CFU/cm^2^ for breast and 5.2 ± 0.0 to 7.4 ± 0.0 log CFU/cm^2^ for thigh depending on the storage temperature. *Pseudomonas* spp. was the dominant microbial group, followed by *B. thermosphacta* and LAB, for all storage temperatures and chicken parts (Figure 1). All spoilage groups were found at higher levels (*p* < 0.05) in the thigh compared to breast at all temperatures, except for microbiota at 10 °C and *B. thermosphacta* at 0 °C, which were found at similar levels. Whereas LAB grew only at 10 °C (4.9 log CFU/cm^2^) by the end of storage in the breast, LAB in the thigh increased at all temperatures and reached populations ranging from 3.9 ± 0.0 log CFU/cm^2^ to 6.6 ± 0.0 log CFU/cm^2^. In the case of chicken breast samples, packaging did not affect the gas composition (i.e., O_2_ and CO_2_ concentrations) throughout storage and, consequently, the development of naturally occurring microbiota. 

**Figure 2:**
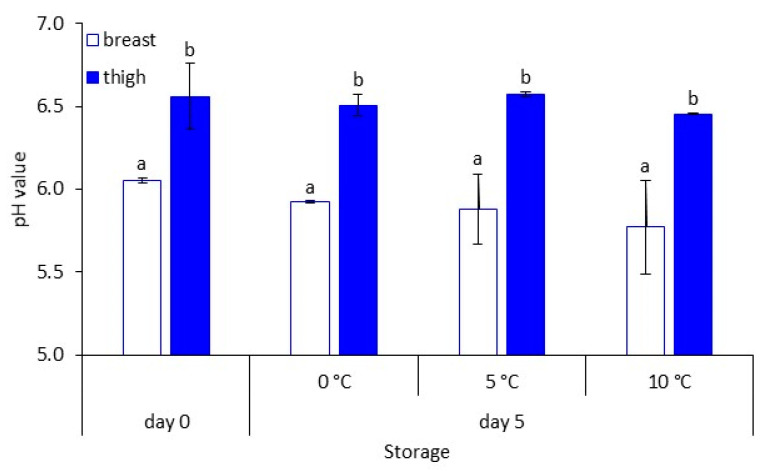
The pH values (mean ± standard deviation, *n* = 2) of chicken breast and thigh fillets at the beginning (day 0) and end (day 5) of storage at 0 °C, 5 °C, and 10 °C. Different letters (a, b) indicate statistically (*p* < 0.05) significant differences.

The pH of chicken breast and thigh at the beginning of storage was 6.05 ± 0.01 and 6.56 ± 0.20, respectively, and remained relatively constant throughout storage presenting minor reductions (Figure 2).

### 3.2. Amplicon Sequencing of the 16S rRNA Gene of Breast and Thigh Fillets 

**Figure 3:**
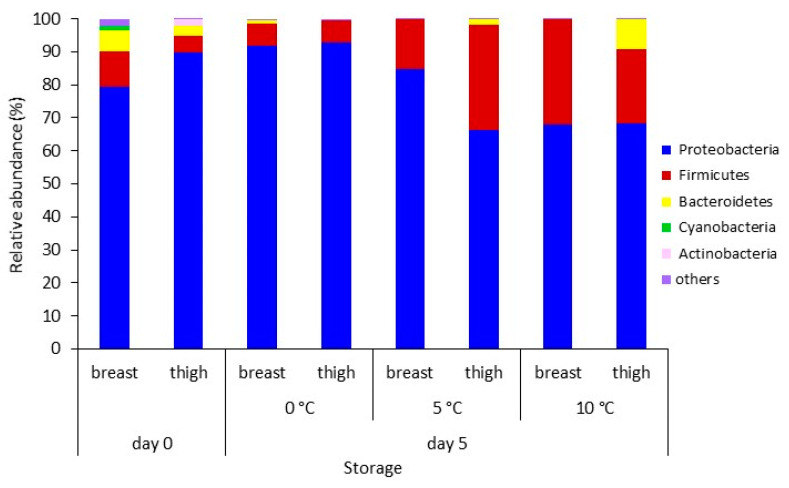
Relative abundance (%) of dominant bacterial phyla on chicken breast and thigh fillets at the beginning (day 0) and end of storage (day 5) at 0 °C, 5 °C, and 10 °C. Phyla with abundance >1% are reported.

To characterize the chicken breast and thigh fillets microbiota at the beginning (day 0) and end (day 5) of storage at 0 °C, 5 °C, and 10 °C, samples were analyzed through 16S rRNA gene amplicon sequencing. Fresh chicken breast and thigh fillets were dominated by Proteobacteria (79.3% and 89.7%, respectively), followed by Firmicutes (11.0% and 5.2%), Bacteroidetes (6.3% and 3.0%), Cyanobacteria (1.4% and 0.1%), and Actinobacteria (0.8% and 1.9%) (Figure 3). Acidobacteria, Planctomycetes, Ignavibacteriae, and Synergistetes constituted other detected phyla (abundance < 1%). At the end of storage, Proteobacteria and Firmicutes phyla remained dominant (total > 90.9%) across all chicken samples and storage temperatures. It seemed that Firmicutes’s abundance increased by the end of storage mainly on thigh stored at 5 °C and 10 °C and on breast stored at 10 °C.

**Figure 4:**
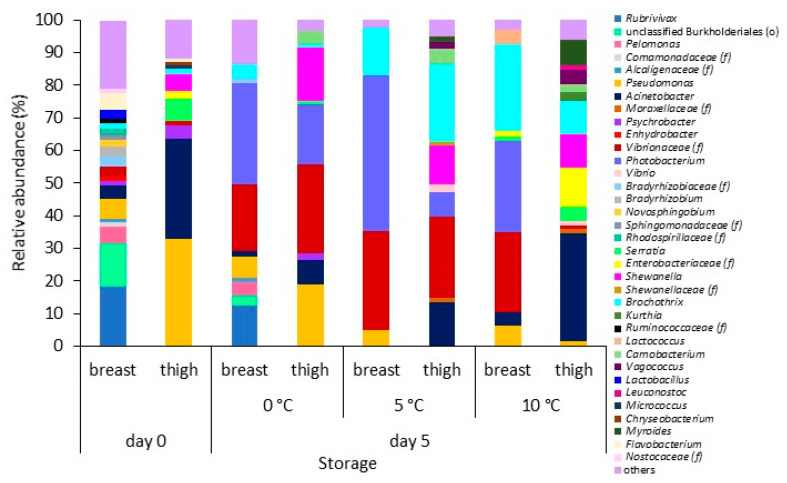
Relative abundance (%) of dominant bacterial genera on chicken breast and thigh fillets at the beginning (day 0) and end of storage (day 5) at 0 °C, 5 °C, and 10 °C. Genera with abundance >1% are reported. f and o in parenthesis indicate family and order, respectively.

A complex initial bacterial microbiota was also revealed at genus level, with a total of 213 different OTUs represented. Relative distributions of the major (>1%) OTUs at the genus level identified on chicken breast and thigh fillets are presented in Figure 4. A total of 143 and 99 genera were assigned to fresh chicken breast and thigh fillets, respectively. The breast fillets microbiota was dominated by *Rubrivivax* (18.3%), unclassified Burkholderiales (order) (13.2%), *Pseudomonas* (6.2%), *Pelomonas* (5.2%), *Flavobacterium* (4.9%), and *Acinetobacter* (4.0%), accounting for 51.8% of the entire genera. In the thighs, the top two predominant genera were *Pseudomonas* and *Acinetobacter*, presenting abundances of 33.0% and 30.4%, respectively. *Serratia*, *Shewanella*, *Psychrobacter*, *Enterobacteriaceae* (family), *Brochothrix* and *Vibrionaceae* (family) were encountered at lower abundances (6.5%, 5.1%, 4.3%, 2.6%, 1.6%, and 1.5%, respectively). 

After storage of breast samples at 0 °C, *Rubrivivax* and *Pelomonas*, both members of the *Comamonadaceae* family, and unclassified Burkholderiales maintained their abundance at lower levels (12.3%, 4.1%, and 3.2%, respectively). At the same time, at the higher temperatures, they constituted an insignificant part of the total bacterial biota (each < 1%) (Figure 4). In contrast, *Photobacterium* (most sequences were assigned to *P. phosphoreum*) and other members of *Vibrionaceae* family appeared to prevail with a total abundance of 51.4%, 77.9% and 52.4% at 0 °C, 5 °C, and 10 °C, respectively, despite their initial (day 0) presence at low abundances (<1% and 3.2%, respectively). Conventional microbiological analyses did not depict their presence through the growth media used in this study for spoilage analysis. *Pseudomonas* and *Brochothrix* (predominantly *B. thermosphacta*), the two main spoilage organisms of muscle tissues, were present at abundances of up to 6.5% (i.e., at 0 °C) and 26.8% (i.e., at 10 °C), respectively, across all temperatures. Their abundance was inconsistent with plate count results as *Pseudomonas* spp. was the dominant bacterial population, followed by *B. thermosphacta*, at all temperatures (Figure 1). *Acinetobacter’s* abundance on most samples was retained at lower levels (i.e., ≤4.1%) over time. *Lactobacillus*, *Bradyrhizobium*, *Novosphingobium*, *Sphingomonadaceae* (family), *Rhodospirillaceae* (family), *Nostocaceae* (family), *Psychrobacter*, *Enhydrobacter*, and *Ruminococcaceae* (family), which initially represented a total of 16.9% abundance, decreased at stored samples (abundance of each < 1%). Conversely, *Lactococcus* and members of the *Enterobacteriaceae* family (predominantly *Serratia*) were detected only at 10 °C (4.3% and 3.1%, respectively). Moreover, LAB represented a small percentage of the total bacterial community at 10 °C (5.1%, mainly *Lactococcus*), which was verified by classical microbiological analysis (i.e., LAB populations remained unchanged at 0 °C and 5 °C until the end of storage, and increased to 5.1 log CFU/cm^2^ at 10 °C) (Figure 1).

In the case of chicken thigh, *Acinetobacter* retained its high abundance after storage at 10 °C (33.0%), while it decreased to 7.3% and 13.6% at 0 °C and 5 °C, respectively (Figure 4). In contrast, *Pseudomonas*’ abundance reduced to 19% at 0 °C, while at 5 °C and 10 °C, *Pseudomonas* constituted a minor part of the microbial consortium (each < 1.4%). This, however, was not in accordance with classical microbiological analyses, since *Pseudomonas* spp. were encountered at levels > 6 log CFU/cm^2^ at the two highest temperatures (Figure 1). Storage at 5 °C and 10 °C increased the abundance level of *Brochothrix* (predominantly *B. thermosphacta*) (24.0% and 10.1%, respectively). Similarly, the storage at 0 °C and 5 °C seems to favor the abundance of *Vibrionaceae* (27.1% and 25.0%, respectively) and *Photobacterium* (most sequences were assigned to *P. phosphoreum*) (18.4% and 7.6%, respectively), while *Shewanella* increased its abundance at all conditions (ranging from 10.1% to 16.4%). Genera belonging to LAB group, i.e., *Carnobacterium*, *Vagococcus*, and *Leuconostoc*, which constituted only a minor (<1%) percentage of the total microbial communities in fresh thigh, increased their abundance at 5 °C and 10 °C, with a total of 6.6% and 8.0%, respectively, which was in accordance with the increase on colony counts obtained from MRS medium. Similarly, *Moraxellaceae* (family) and *Vibrio* increased their abundance up to 1.5% and 2.2%, respectively, at the same temperatures. On the other hand, *Enterobacteriaceae* (family), *Myroides*, and *Kurthia* increased their abundance to 12.0%, 8.1%, and 2.9%, respectively, at the highest temperature. *Serratia* was present at 0 °C and 10 °C at lower abundance levels than at the beginning of storage. 

**Figure 5:**
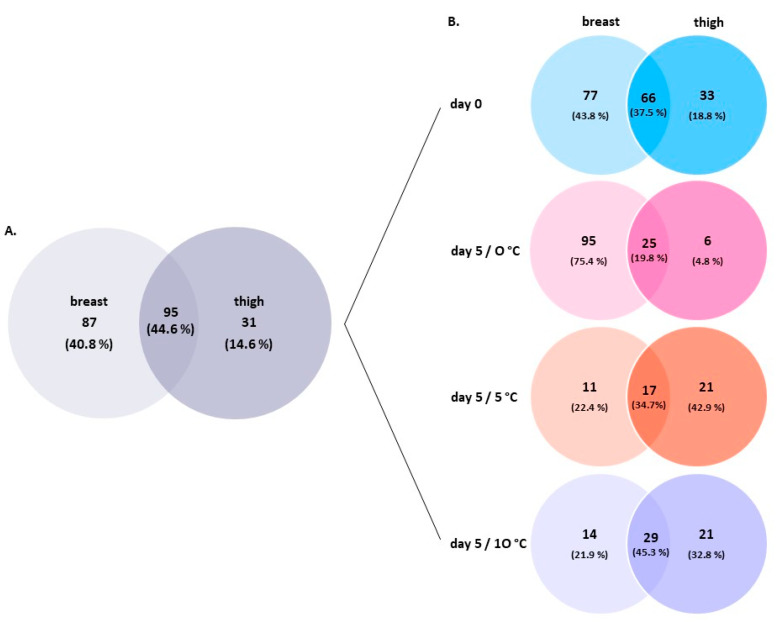
Venn diagram demonstrating unique and shared Operational Taxonomic Units (OTUs) at the genus level between chicken breast and thigh fillets based (**A**) on total microbiota and (**B**) on microbiota deriving from each storage condition.

To elucidate the nature of the shared (core) versus unique (satellite) bacterial microbiota by product type (i.e., breast and thigh fillets), a Venn diagram was constructed (Figure 5). Chicken breast fillets presented higher bacterial richness and unique OTUs at the genus levels than thigh fillets (87 and 31 OTUs, respectively). A core group with 95 (44.6%) taxa were common to both chicken parts (Figure 5A). At the beginning of storage, fresh (day 0) chicken breast and thigh fillets shared 66 (37.5%) OTUs. Within this core group, six (24.0%) genera, including *Acinetobacter*, *Brochothrix*, *Flavobacterium*, *Pseudomonas*, *Psychrobacter*, and *Vibrionaceae* (family), were the most abundant (each >1%) (Figure 5B). At the end of storage, the taxonomical structures of bacterial OTUs became less diverse at all temperatures and chicken parts. Breast and thigh samples shared 25 (19.8%), 17 (34.7%) and 29 (45.3%) bacterial genera at 0 °C, 5 °C, and 10 °C, respectively (Figure 5B). Among the core microbiota, the most abundant (each >1%) at 0 °C included the *Acinetobacter*, *Brochothrix*, *Photobacterium*, *Pseudomonas*, and *Vibrionaceae* (family); at 5 °C, the *Brochothrix*, *Photobacterium*, and *Vibrionaceae* (family); and at 10 °C, the *Acinetobacter*, *Brochothrix*, *Enterobacteriaceae* (family), *Pseudomonas*, *Serratia*, and *Vibrionaceae* (family). More unique bacterial genera were accounted for breast fillets (95 OTUs) stored at 0 °C compared to thigh fillets (6 OTUs), while the opposite was observed at 5 °C (11 OTUs and 21 OTUs, respectively) and 10 °C (14 OTUs and 21 OTUs, respectively) (Figure 5B). 

**Figure 6:**
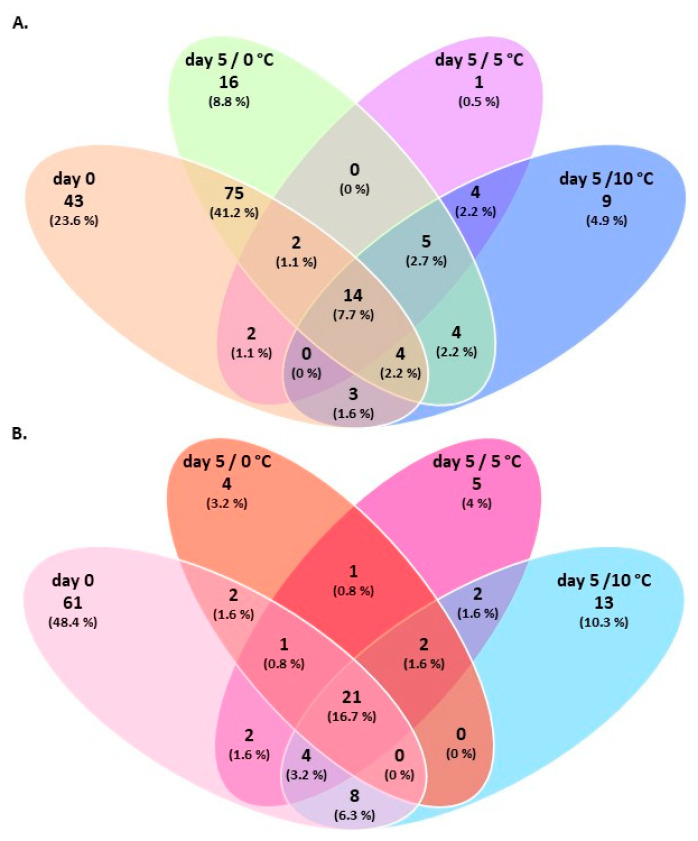
Venn diagram demonstrating unique and shared Operational Taxonomic Units (OTUs) at genus level within (**A**) chicken breast and (**B**) thigh fillet samples at the beginning (day 0) and end (day 5) of storage at different temperatures.

Similarly, a Venn diagram was constructed, showing the overlap of genera within each chicken part over time (Figure 6). Chicken breast samples shared 14 (7.7%) groups across storage, with three (12.5%) assigned to *Brochothrix*, *Pseudomonas*, and *Vibrionaceae* (family) being the most abundant (total 12.5%) (Figure 6A). *Photobacterium* was also common among samples at the end of storage at 0 °C, 5 °C, and 10 °C. In the case of thigh fillets, 21 (16.7%) bacteria were commonly detected within samples, with *Acinetobacter*, *Brochothrix*, *Shewanella*, and *Vibrionaceae* (family) being the most abundant (total 20%) (Figure 6B). In addition, *Carnobacterium* was also detected in samples after 5 days of storage at all temperatures. 

**Figure 7:**
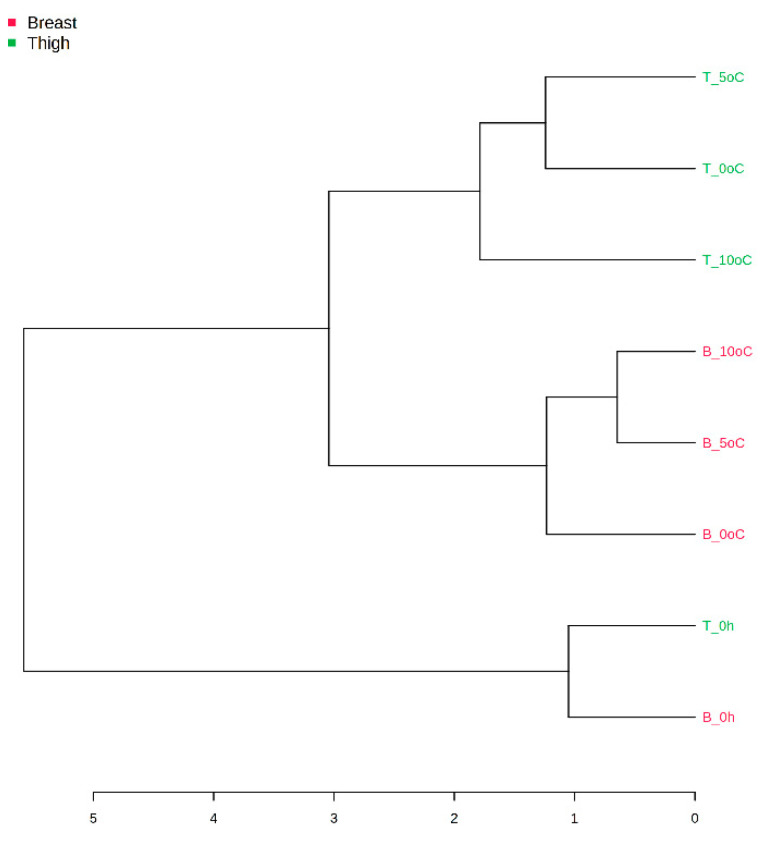
Hierarchically clustered dendrogram of bacterial Operational Taxonomic Units (OTUs) at the genus level from chicken breast (B) and thigh (T) fillets at the beginning (0 h) and end of storage at 0 °C, 5 °C, and 10 °C. Clustering was performed using Pearson’s correlation coefficient and Ward’s linkage.

To further explore the correlation of chicken parts and detected microbiota, a PLS-DA of the entire genus-level OTUs was conducted. The analysis showed discrimination of samples between the chicken cuts, with no overlapping. However, this discrimination was not statistically confirmed (Figure S1). Moreover, the hierarchical clustering dendrogram revealed that samples were clustered depending on storage time (i.e., 0 and 5 days) and chicken part (i.e., breast and thigh fillets) (Figure 7).

## 4. Discussion

In this study, the bacterial composition of chicken breast and thigh fillets and changes in richness and abundances after storage at refrigeration (0 °C and 5 °C) and slightly abuse (10 °C) temperatures were explored using NGS technology along with the conventional plate count method. 

Microbial enumeration revealed that TVC of chicken breast and thigh fillets were found at relatively low levels and within the bacterial loads usually reported in the literature for chicken and other poultry meats [7,51,52,53,54,55]. Briefly, the initial microbial biota of both chicken parts consisted mainly of *Pseudomonas*, *Br. thermosphacta*, and LAB. By the end of storage, the microbial consortium on both chicken parts was dominated by *Pseudomonas*, followed by *B. thermosphacta* and LAB [7,56,57] at similar population levels between the different parts, especially at the highest storage temperature. As expected, breast muscle pH was lower than the thigh, since the breast has been shown to contain higher amounts of glycogen and therefore lactic acid [54,58]. Throughout storage, pH values remained relatively constant [51,57]. 

During the last two decades, the evolution of NGS, such as 16S rRNA gene Ion Torrent amplicon sequencing, has enabled a deep understanding of the foods (including chicken)-related microbiome without the limitations of conventional cultivation methodologies. Although partial 16S rRNA NGS does not necessarily provide an absolute quantification of microbial abundances [59] and/or achieve the phylogenetic resolution offered by the sequence of the entire 16S rRNA gene [60], it seems to indicate how bacterial taxa change over time at different storage conditions and on different meat parts [25,35,40,61,62]. In this study, the metataxonomic analysis revealed the discrimination of the characterized microbial community profiles based on the chicken part and storage time. Factors such as the initial microbiota, processing practices, structural and compositional characteristics of the chicken parts, and storage temperature appear to shape the chicken meat microbiome, and, consequently, the evolution of spoilage, differently [13,39,42,63,64]. In brief, the 16S rRNA metataxonomic analysis performed in this study revealed a complex taxonomical structure for both chicken breast and thigh fillets, including >200 OTUs at the genus level. In the case of fresh (day 0) chicken parts, a total of 176 genus-level OTUs were identified. As expected, the increase of storage temperature and the passage of time seemed to reduce the bacterial richness, since 126 OTUs, 49 OTUs, and 64 OTUs were identified at 0 °C, 5 °C, and 10 °C, respectively. This observation suggests that a fraction of the total bacteria population dominated and was responsible for spoilage [13,39,65]. The core microbiota of chicken breast and thigh fillets at the end of storage was dominated by Proteobacteria and followed by Firmicutes. The genera *Acinetobacter*, *Photobacterium*, *Pseudomonas*, and members of *Vibrionaceae* family within the Proteobacteria phylum and *Brochothrix* within the Firmicutes phylum occurred in >80% of the total detected sequences per sample. It needs to be mentioned here that this depiction of microbiota was not totally in line with the results obtained by conventional microbiological analysis. It is worth noting that NGS analysis highlighted the occurrence of bacterial families and genera that were not specifically enumerated by the applied culture plating (i.e., *Acinetobacter* and genera of *Vibrionaceae* family, e.g., *Photobacterium*). An explanation of this situation could be attributed to the limitations of the cultured methods [14], which are specifically discussed below for the different microbial groups. 

*Pseudomonas* has been recognized as a predominant psychotropic meat spoiler, owing to the production of proteolytic and lipolytic enzymes, biosurfactants, and pigments [10,14,15,40,42,66,67,68,69,70,71]. Surprisingly, despite their widespread distribution within the poultry chain [67,71] and the dominant population levels depicted in this study by the plate count technique, *Pseudomonas* were only found at high abundances (33.0%) on fresh thigh fillets. On the contrary, *Pseudomonas* presented relatively low abundances on spoiled or semi-spoiled chicken samples, ranging from <1% to 19.0% (depending on the storage temperature and chicken part). The differences between the *Pseudomonas* abundances observed herein and those reported in the literature for chicken meat [40,42] can probably be attributed to the outgrowth of competing bacteria, including *Brochothrix*, *Acinetobacter* and/or members *of Vibrionaceae* family. Another possible explanation could be the ineffectiveness of the culture medium typically used for enumeration of pseudomonads on foods to actually select only for pseudomonads, thus leading to misleading outcomes concerning the real population levels [72,73]. 

On both chicken breast and thigh fillets, *Vibrionaceae* was one of the most abundant families identified, ranging from 2.4% to 78.5% depending on the storage time, temperature, and chicken part. *Photobacterium* is one of the eight genera comprising the *Vibrionaceae* family, and its presence at significant abundances (i.e., from <1 to 47.7%) was evident on most samples. This finding is consistent with other amplicon sequencing studies on various meats, including chicken, turkey, beef, and pork, irrespective of the packing conditions (i.e., air, modified atmosphere, and/or vacuum) [61,74,75,76,77,78,79,80]. *Photobacterium* is widely associated with marine habitats [81] and, until recently, has been broadly considered as a potent spoiler of fish and seafood products [13,82,83,84]. However, in a recent metatranscriptomic analysis, it was proposed that *Photobacterium* spp. are responsible for producing various potent spoilage metabolites on meat, including biogenic amines [75]. Major representatives of *Photobacterium* in meats have been assigned to the species *P. phosphoreum*, which was also depicted by the findings of the present study, *P. iliopiscarium* and *P. carnosum* [78,79]. However, it was mentioned before that the identification of *Photobacterium* species could not be accomplished due to the low discriminatory power of 16S rRNA gene within the genus *Photobacterium* [79]. Their occurrence in raw fresh or spoiled meats is yet underestimated or not detected by conventional microbiological methods as photobacteria are psychrotrophic, nutritionally fastidious, and require sodium chloride for growth, and usual (non-)selective culture media used for determination of spoilage bacteria hinder their growth [78,85]. In this study, *Photobacterium* enumeration was not considered. However, the detection of *Photobacterium* sequences by NGS analysis suggests, again, that the selective medium for isolating and cultivating this organism [79] must be employed in future studies dealing with meat spoilage.

*Acinetobacter*, belonging to the *Moraxellaceae* family, was detected on most chicken samples despite the observed differences in detection abundances. Although is a strictly aerobic, Gram-negative microorganism, its occurrence on fresh and spoiled meats under aerobic, modified atmosphere and vacuum-storage conditions is well documented [14,37,42,61,86,87,88,89]. The proportion of *Acinetobacter* decreased on all chicken samples stored at 0 °C and 5 °C or remained stable on thigh samples stored at 10 °C. This could be attributed to the relatively high optimal growth temperature of *Acinetobacter* (i.e., from 20 °C up to 44 °C, depending on strain) [86,90,91]. *Psychrobacter*, also belonging to the *Moraxellaceae* family, was detected as a small part of the initial microbiota. *Acinetobacter* and *Psychrobacter* are considered mild spoilers of meat and dairy products, considering their lipolytic activity and lack of proteolytic activity [92,93].

*Shewanella*, a genus closely related to *Pseudomonas*, was among the most common genera detected in chicken thigh samples. This genus is considered the dominant spoilage organism of iced stored fish due to the production of volatile sulfides and trimethylamine [94]. Nevertheless, its presence on fresh and spoiled broiler chickens and products is well documented [14,40,41,63,95].

*Brochothrix* (*Listeriaceae* family) is Gram-positive organism that is ubiquitous in meat production chain and has been frequently isolated and recognized as a common spoilage species on fresh and on modified atmosphere and/or aerobically cold-stored meats [76,96,97,98,99,100]. On both chicken breast and thigh fillets, the genus was represented at the species level by *B. thermosphacta*, and its abundance was increased at stored samples. This observation is in accordance with the relevant increase in population levels observed by conventional plate counting. *B. thermosphacta* produces a wide variety of compounds such as acetoin, acetic, 2,3-butanediol, fatty acids, and alcohols, imparting cheesy odors responsible for a distinct type of meat spoilage [10,101,102]. 

LAB seemed to constitute only a minor part (mainly at 10 °C) of the total bacterial community at the end of storage. The classical microbiological analysis also verified this observation. *Carnobacterium* (represented by *C. maltaromaticum*), which constituted the core psychrotrophic LAB microbiota on thigh samples, has been frequently isolated from chilled poultry [34,37,41]. However, its contribution to meat spoilage has been reported to be negligible due to the production of volatiles with low sensory perception [65].

With particular reference to poultry food safety, *Salmonella* and *Campylobacter* genera, two pathogenic bacteria often identified on chicken carcasses and products, were not detected on breast and thigh fillets via microbiome analysis nor detected with extremely low abundances (i.e., *Campylobacter* sequences of 0.05% in fresh thigh). These results are in agreement with previous studies [35,40]. Additionally, *Arcobacter* and *Helicobacter*, two potential foodborne pathogens assigned to the *Campylobacteraceae* family [103,104], were detected on fresh chicken samples at low abundances (<1%). Moreover, *Serratia*, *Myroides* and *Vibrio* were identified at considerable abundances (ranging from 1.2% to 8.1%) on thigh fillets at the end of storage at 5 °C and/or 10 °C. Species within these genera may contribute to food spoilage but could also be considered as opportunistic pathogens [105,106,107,108,109], indicating the need for more effective interventions to control pathogens in the processing environment.

## 5. Conclusions

In this study, the microbial diversity of two commonly consumed chicken fillets was examined at refrigerated and slight abuse storage temperatures to explore the differences between the different chicken parts and storage temperature. The amplicon-based NGS of the 16S rRNA gene revealed the microbial communities that inhabit chicken breast and thigh fillets. Storage increased the microbial loads and decreased the taxonomic richness and abundances on both chicken parts, indicating that a subset of bacteria became dominant, which was more evident with increasing temperature. *Brochothrix* and LAB seemed to be more associated with storage at 5 °C and 10 °C, while genera of *Vibrionaceae* family, including *Photobacterium*, were more associated with storage at 0 °C and 5 °C. Moreover, the potential of different chicken parts to harbor different bacterial populations was also highlighted. NGS analysis verified the occurrence of bacterial families and genera (e.g., *Acinetobacter* and *Photobacterium*) that could not be detected by culturing and growth media routinely used in spoilage studies. Thus, this study provides complementary information on the core microbiota encompassing putative spoilers of chicken meat.

## Data Availability

Data reported in this manuscript will be available upon request.

## Supplementary Materials

The following are available online at https://www.mdpi.com/article/10.3390/foods10040765/s1, Figure S1: Partial least squares discriminant analysis (PLS-DA) clustering of chicken samples depending on chicken part (i.e., breast and thigh fillets).
