# Supplementary material for: Microbiota of Chicken Breast and Thigh Fillets Stored under Different Refrigeration Temperatures Assessed by Next-Generation Sequencing
Source: Foods. 2021 Apr 3;10(4):765. doi: 10.3390/foods10040765 (PMC8066510; doi:10.3390/foods10040765)
Supplement: Supplementary file 1 [file foods-10-00765-s001.pdf]

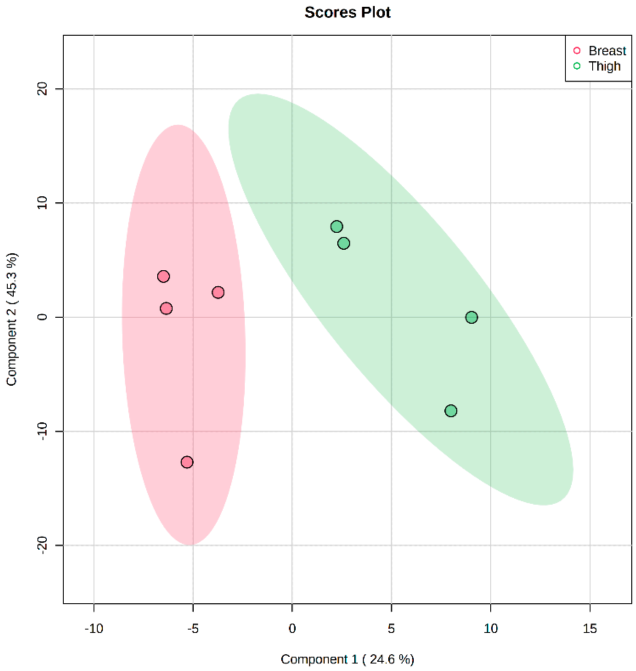


**Figure S1.** Partial least squares discriminant analysis (PLS-DA) clustering of chicken samples depending on chicken part (i.e. breast and thigh fillets).
